# Parents’ lived experience of early risk assessment for cerebral palsy in their young child using a mobile application after discharge from hospital in the newborn period

**DOI:** 10.1080/07853890.2024.2309606

**Published:** 2024-02-01

**Authors:** Annemette Brown, Åsa B. Tornberg, Inger Kristensson Hallström

**Affiliations:** a Department of Health Sciences, Faculty of Medicine, Lund University, Lund, Sweden; b Department of Pediatrics and Adolescence Medicine, Nordsjælland University Hospital, Capital Region of Denmark, Denmark; c Department of Neurology and Physiotherapy, Nordsjællands Hospital, Capital Region of Denmark, Denmark

**Keywords:** Cerebral palsy, experiences, general movements, mobile application and parents

## Abstract

**Introduction:**

General Movement assessment (GMA) is considered the golden standard for early identification of infants with a high risk of developing cerebral palsy (CP). The aim of this study was to explore parents’ lived experience of early risk assessment for CP using a mobile application for home video recording after discharge from hospital stay in the newborn period.

**Methods:**

An inductive qualitative design using a hermeneutical phenomenological approach was chosen, and fourteen parents with children at risk of CP were interviewed at home. The hermeneutical phenomenological approach describes humans’ lived experiences of a specific phenomenon with a possibility of deeper understanding of the expressed statements. The interviews were analyzed using the fundamental lifeworld existential dimensions as guidelines for describing the parents’ lived experience.

**Results:**

The overall understanding of the parents’ experience was ‘Finding control in an uncontrolled life situation'. During the often-long hospitalizations, the parents struggled with loss of control and difficulty in understanding what was going on. The use of the mobile application followed by a swift result made them feel in control and have a brighter view of the future.

**Conclusions:**

The findings suggest that the mobile application did not seem to worry the parents. Instead, it provided the parents with a sense of active participation in the care and treatment of their child. The mobile application should be accompanied with clear instructions and guidelines for the parents and details about how and when the result is given.

## KEY MESSAGES

For the first time, parents’ experiences concerning early assessment for cerebral palsy using a mobile application are profoundly explored.

Early risk assessment for cerebral palsy performed by parents at home using a mobile application did not seem to increase the parents’ worry; instead, it gave them a sense of control.

Involving parents in the care and treatment of their child is vital to increase parental participation and control.

## Introduction

Globally, infants with perinatal brain injury represent more than nine million years lived with disabilities [1]. Functional consequences of perinatal brain injury will often appear months or even years later, which will delay therapeutic intervention and result in uncertainty about the child’s health status for a long period. Around 15–20% of Danish children born very preterm (<32 weeks of gestation), are diagnosed with a broad range of neurodevelopmental impairments [1], and approximately 5% have the characteristics of cerebral palsy (CP), some children are not diagnosed until the age of five years [2]. CP defines as ‘a non-progressive neurological disorder affecting multiple motor systems as muscle control and tone, feeding and swallowing difficulties as well as balance and coordination’ [3]. In Denmark, 125–150 [4] children are diagnosed annually with CP. Clinically the diagnosis is first established when the children show firm signs of CP. In Denmark 52% of the children are diagnosed at the age of 18 months, and a small share of them are diagnosed between the ages of 4 and 5 years [2,5,6].

There is a growing body of evidence that early intervention improves functional outcomes of infants at risk of neurodevelopmental impairment [4,7]. However, it is a challenging task to identify children who are at the highest risk of neurodevelopmental impairment at an early age [8]. General movement assessment (GMA) is a non-invasive observational method used to describe the infant´s spontaneous gross motor motility in relation to the maturation of the brain. General movements are observable from fetal life to approximately 5 months post term age [9]. The accuracy is highest when GMA is performed during the fidgety movement period at 10 to 20 weeks post-term age [10]. The method is based on Gestalt perception [10] and is a non-invasive method in which the child’s spontaneous motor movements are recorded on video, after which a GM observer [10] classifies the quality of GM movements in different categories. These movements are a strong marker for later developmental disabilities, CP, especially seen in the so-called ‘Absent Fidgety Movements’ that occur when the child is aged 9–20 weeks post term. It is important to emphasize that using GMA for early risk assessment for CP is not an assessment for CP diagnosis but only a risk estimate and therefore the assessment should be handled with caution [8,11,12].

In person GM assessment is time-consuming for the child, its parents, and GMA trained personnel, and the method requires the parents to travel with their young and fragile child to the hospital for testing [12]. Mobile technologies have made it possible to develop mobile applications that could be available for many people independent of region, country, and continent. Free et al. 2013 [13] demonstrated that mobile technology interventions, such as mobile applications, improved healthcare delivery and had an advantage over other communications technologies. A broader use of home-based video recording to be objectively analyzed and processed by newly developed software as the Baby Moves mobile application [14] may make it possible to focus hospital follow-up resources and provide targeted interventions at an early age to those infants at the highest risk of developing CP in a regional, national, and global context. Hence a recent multicenter international feasibility study testing home-based video recording using a mobile application has demonstrated that home-based video recordings are feasible for 97% of families [15].

Giving birth to a healthy child is a life event for the parents and their family. Giving birth to an unexpectedly sick or premature child often replaces excited emotions with anxiety, distress, and worry [16]. Children born with severe complications and/or prematurity are admitted to neonatal intensive care units, which is extremely stressful. The parents worry about whether their child is going to survive and later they worry about their child’s future life [17]. Introducing new technology such as a mobile smartphone application to screen for risk of CP in infants with a complicated start in life might however concern the parents in both the short and the long term. Parents’ experience, as demonstrated by Kwong et al. [14], showed that they appreciated being able to video record their child at home instead of in a hospital setting. So far, however, no research has explored parents’ experience of early risk assessment of using GMA in a home setting.

Therefore, the aim of this study was to explore parents’ lived experience of early risk assessment for CP using a mobile application for home recording after discharge from hospital stay of the newborn period.

## Method

### Design

A descriptive inductive design with a hermeneutic phenomenological approach based on van Manen [18,19] was used to describe parents’ lived experience. Hermeneutic phenomenology is a descriptive methodology where lived experience is recollected after it is passed or lived through. The hermeneutic phenomenological approach is to go beyond what is said and to understand the underlying experience of what has been lived through by transforming the lived experience into a textual expression of its essence [18,19]. As described by van Manen the four fundamental lifeworld existentials; ‘lived body', ‘lived space', ‘lived time', and ‘lived others’ were used to describe, clarify and deepen the understanding of the participants’ lived experiences [18,19].

### Procedure

The study took place in the northern part of the Capital Region of Copenhagen in Denmark. Qualitative interviews in the homes of the parents were performed from 15 March 2018 until 21 February 2019.

Fourteen Danish parents were strategically selected from the multicenter feasibility study ‘In-Motion-App for remote General Movement Assessment: a multi-site observational study’ [15], based on socioeconomic background, civil status, age, and academic background. The parents were asked prior to discharge if they were willing to participate in an interview concerning their experiences of early risk assessment for CP using the In-Motion mobile application after they have used the device. Inclusion criteria for the study were: Parents of infants with high-risk of perinatal brain injury based on [1] Birth weight ≤1000 g and/or gestational age < 28 weeks [2]; Neonatal arterial ischemic stroke [3]; Neonatal encephalopathy [4]; Other significant risk factors such as intrauterine growth retardation, perinatal hypoglycemia and infants born with withdrawal syndromes.

#### In-motion mobile application

The In-Motion mobile application was developed for parents and health care personnel to record a video of the infant’s spontaneous movements during the fidgety period [15]. Each infant was given a unique ID, and the infant’s date of birth and expected date of birth were registered in the app. In the application the parents got an instruction video in Danish providing information and brief instructions on positioning of the infant, how to use their smartphone, and how to upload the video.

The parents were instructed to video record their child twice. The first time when the child was between 12 + 1 to 13 + 6 weeks post term and the second time between 14 + 1- and 17 + 6-weeks post term age. They were reminded by notifications from the study coordinator when to record the videos. When the video was uploaded successfully, they received a message through the application [15].

### Participants

All invited fathers and mothers agreed to participate. The parents included two single mothers and 12 mothers and 12 fathers living together, with a total of 18 children. 13 parents were under the age of 30, 11 were between 30 and 49 years of age, and two parents were over 50 years. Seven parents (four fathers and three mothers) had an academic background of 12 years of education, 17 parents (seven fathers and 10 mothers) had an academic background of more than 12 years of education, and the remaining parents had less than 12 years education and 11 parents were first-time parents. Eight children were born before gestational age of 28 weeks and six children were twins, with a birthweight below 1000 g and they spend more than 2 months in neonatal intensive care unit (NICU). Four children were born before 32 weeks and with a birthweight of less than 1500 g, they spend less than six weeks in NICU. Four children born at full term where three children had neonatal encephalopathy and underwent hypothermia and one child suffered neonatal arterial ischemic stroke, all four had a birthweight above 3500 g and were discharged within two weeks. Two children were born at full term, one with symptomatic perinatal hypoglycemia and one child was born with growth retardation more than 50% with a birthweight below 1500 g. These two children spend a month at the NICU before being discharged.

#### Data collection

Prior to the interview the parents had received their child’s GMA result in person by their local neonatal physiotherapist. The result was given to the parents within 14 days from the day they uploaded their second video. Within 14 days after they had received the GMA result, they were given an appointment with the child’s neonatologist and pediatric physiotherapist at the developmental outpatient clinic of the local hospital.

The first author interviewed the parents at a time suitable for them; 13 interviews were performed in Danish and one in English. Efforts were made to ensure that both parents (if relevant) could be present during the entire interview.

An interview guide with open-ended questions based on earlier research associated with perinatal and early infant screening was used [8]. The first two interviews were used as pilot interviews before continuing with the rest of the interviews. As no major changes were made to the interview guide the pilot interviews were included in the analyses. Before the interview started, the interviewer used some conversation time to make the parents feel at ease. Each interview started with the parent/parents being asked to narrate in their own words the birth and their stay in hospital by the question: ‘How did you experience the birth of your child and the following stay at the neonatal ward? ‘ Questions such as ‘Can you explain further? ‘, ‘Can you explain how that made you feel? ‘, and ‘Can you please elaborate on that particular emotion? ‘ were asked for clarification and to get the perspectives of both fathers and mothers and to create space for both parents to express themselves. The next questions asked were: ‘How did you experience the information of the mobile application? In particular, what did you think of the use of the word cerebral palsy in the information leaflet? ‘, ‘How did it make you feel? ‘ and ‘How did it make you feel to record and send the video *via* the mobile application? ‘ Finally, parents were asked about receiving the result from the video and the waiting time; ‘How did it make you feel when you had the video for assessment? ‘, ‘How did you experience the waiting time for the result? ‘

The interviews took 60–90 min and lasted until the parents themselves stated that they had nothing further to add. All the interviews were audiotaped on an iPhone mobile application, transcribed verbatim by the first author, and stored on a secure server at Nordsjællands Hospital, Denmark.

After each interview the interviewer wrote field notes about emotions during the interview, eye contact, unrest, changing focus and other emotional manifestations. Each interview was given an identification number, and names, places, and professionals appearing in the interviews were pseudonymized. Quotes were used to illustrate the parents’ lived experience and professionals as well as family members were anonymized in the text and described as nurse, physician, sibling, or midwife.

Data saturation seemed to appear after 12 interviews but additional two interviews were made to assure that no new information appeared.

### Ethical considerations

The study was approved by the Danish Committee System on Health Research Ethics, reg.no. 170117778 and by the Danish Data Protection Agency. NOH-2017-025, I-Suite no. 05988. The study followed international guidelines outlined in the Declaration of Helsinki [20]. Written consent was obtained from all parents. Confidentiality was explained in an information leaflet and orally prior to consent. Parents were told that they could withdraw from the study if they wanted. To avoid any obligation on the part of the parents, a designated nurse at the neonatal ward included the parents in the study. All parents could be referred to the ward counselor for talks if necessary.

### Data analysis

By active listening and reflecting during and after each interview a thematic analysis guided by van Manen’s (1997) [18,19] hermeneutic phenomenological approach started in parallel with the data collection by the first author. Each transcribed interview was read by all the authors to get a naïve understanding and to capture the overall meaning of the parents’ lived experience. Thereafter the first and last author read six interviews independently, and the second author read four, scrutinizing every sentence and asking what it showed about the parents’ lived experience. Discussions were held when the first and last authors reflected upon their understanding of the parents’ lived experience. The first author then read the remaining eight interviews independently in the same thorough way and reflected on the meaning of the parents’ lived experience. Key sentences revealing the parents’ lived experience were highlighted and color-coded to describe the four fundamental dimensions of lived body, lived time, lived space, and lived human relations in all interviews. The four fundamental dimensions were used to explore the parents’ lived experience as it appeared when the parents gave their narratives by an inductive approach. The analysis moved between the four lifeworld existentials, (the parts), and the overall understanding (the whole) through reflective writing and rewriting as described by van Manen [18,19] to transform the essence of the participants’ lived experience into text.

Through close collaboration, reading of interviews, attention to field notes and discussions between the first and third authors [18,19] preliminary subthemes emerged describing the parents’ experience. Thereafter, the second author read the coded material, and discussions between all three authors were used to develop a nuanced description of the parents’ lived experience based on the four lifeworld existentials. The field notes were used to gain an in-depth understanding of the parents’ experiences of home-based early risk assessment for CP by means of a mobile application. During reflections it became evident that the parents’ experience of the risk assessment was only one part of the parents’ experience. To describe the uniqueness and significance of the parents’ experience it was important to also include their overall experience of their child’s birth and transition to home. The first and second authors then continued the reflective writing and rewriting process according to the hermeneutical phenomenological process [18,19] which included regular discussions between all authors about the parts and the whole. As subthemes and themes emerged, they were organized and refined within each lifeworld existential ending in an overall theme and three subthemes describing the parents’ lived experience [18,19].

Only the first author coded all the interviews. Then the second author coded four interviews and the last author coded six interviews, to validate the latent interpretation of manifest transcripts of the first author by triangulation.

No themes were derived in advance. Instead, the themes were constructed during the analysis process and according to the phenomenological hermeneutic methodology. After the subthemes were identified a theme summarizing the parents’ overall experience was described.

## Results

The overall understanding of the parents’ lived experience was described in one essential theme, ‘Finding control in an uncontrolled life situation', based on three themes and nine subthemes (see Table 1). The parents found themselves in an alien situation and had no previous experiences of what they were facing. For long times they were in shock, scared, worried, and unhappy. The early risk assessment for CP using a mobile application was described as a small and simple task that strengthened them in the endeavor to overcome past difficulties. The parents described struggling with a challenging start to their parenthood, which formed the first theme ‘A challenging start to parenthood'. During the often-long hospitalization they felt as if they were living beside reality, which formed the second theme, ‘Living in a bubble'. The parents indicated that they did not feel in control of their lives until they came home from the hospital, and this formed the third theme, ‘Getting control in a safe environment'. Then they saw that their child made improvements, and by means of the app believed in their own ability to see their child’s progress.

**Table 1.** Overall theme, themes and subthemes.

Finding control in an uncontrolled life situation		
A challenging start to parenthood	Living in a bubble	Getting control in a safe environment
Difficult pregnancy and delivery	Difficulty in processing emotions	Feeling safe at home
Difficulty in coping with highly specialized care	Difficulty in processing information	Can see that the child is developing
Separation and difficulty in bonding with the newborn child	Handing over responsibility to the experts	The app gives control and security

### 'A challenging start to parenthood'

This theme describes a challenging start to the parents’ parenthood. Unprepared, they were thrown into an uncontrolled life situation, which often started with a complicated pregnancy followed by traumatic delivery. When the child was born, they suffered from their child’s unstable medical condition and the uncertainty of not knowing whether their child would survive. They found it difficult to bond with their newborn child when struggling to cope and find control in the highly specialized care their child ended up in. When they learned to deal with the routines in the highly specialized setting, they were moved to their local neonatal hospital with new routines and new uncertainties. Slowly, they felt that they got to know their child, became parents, and could start their parenthood.

#### Difficult pregnancy and delivery

The parents described with great emotions the – in many ways – difficult and traumatic start of their parenthood. They described challenges during many years of longing and failing to become pregnant, with miscarriages and repeated and difficult artificial In-Vitro-Fertilization (IVF) treatments. These treatments were hard for both parents and affected their mental and physical health. This almost made them give up their dream of becoming parents, and then they experienced it as a miracle when an artificial IVF treatment actually worked. They described complication during pregnancy with long periods of bedrest for the mother, for weeks to months at home or in hospital to prevent premature labor, which made them feel anxious and out of control.

I knew it was my last chance of becoming a mother, I had already decided that this was my last IVF treatment, when the pregnancy became complicated, I prayed that it was going to be okay (ID 5209).

Uncomplicated pregnancies sometimes became complicated when the mother felt less intrauterine life and ended up with acute cesarean section or when the pregnancy resulted in an acute delivery with a premature birth. Complicated delivery resulted in the child being taken for intensive treatment, which made the parents anxious and fear for the child’s survival. One or both parents were separated from the child for shorter or longer periods due to the severity of the child’s condition. Mothers found the time after the delivery traumatizing when they lost contact with their newborn child and their partner, when their child was moved to intensive care. They experienced the time alone, waiting and lacking comfort from anyone.

This was the wildest rollercoaster I have ever been on. At first it made me physically sick, I had to go to the restroom to collect myself. The staff got very anxious, thought I was doing drugs, but I just had to collect myself and get the strength to be there for my child and wife (ID 5209).

#### Difficulty in coping with highly specialized care

In the highly specialized care, the parents felt anxious, angry, and powerless. These feelings increased when different staff members gave different information and communication about their child’s state and development. They lacked information about how to care for their child as parents, which made them feel helpless and not know what was expected of them, like spectators in their own play. The parents described a difficult time as their sick child was moved between different wards and highly specialized and local hospitals. They lacked information about the transfer and what was expected for the care of their child. When they were unfamiliar with the child’s treatment plan, they became nervous and anxious.

Being admitted to neonatal intensive care hospital was extremely daunting because you follow how all the admitted children were by looking at the monitor in the room. When we were there, two children died on the same day, and I saw it all on the screen. After that we never left our child alone with the staff (ID 5522).

#### Separation and difficulty in bonding with the newborn child

The parents felt that their life situation became difficult as their child was fragile and often in an incubator, closely monitored by highly specialized technology and needing help to breathe. The separation from their child felt unbearable and it was hard for them to believe that their critically sick child would survive when they could not be physically close to their child.

My child was critically ill; I didn’t get a chance to hold her. The child needed emergency hypothermia treatment and was transferred to the neonatal intensive hospital. My husband accompanied the child, and I followed hours later. Due to treatment, I was unable to hold my child and my distress was immense as I believed the child would not survive or would get serious brain damage, I was crying non-stop (ID 9913).

### 'Living in a bubble'

The parents felt as if they were living in a world of their own. Having to stay there for months, the hospital room became their home. Being tired, sad and confused, it was difficult for them to process their emotions and the information given by the professionals. Confidence in the healthcare system and the support from the healthcare staff saved them from total despair.

#### Difficulty in processing emotions

Feeling insecure and without control made it difficult for the parents to deal with their emotions. Parents found it difficult to see their child connected to technical equipment, not knowing if their child was alive. They had difficulties following the progress of their child and they did not comprehend or understand their child’s progress. They saw their child as fragile and critically ill even though the discharge day was getting closer.

When they were able to be physically close and hold their child, they were insecure and did not know if they handled their child in the right way. They felt happy as they could be close skin-to-skin and scared of doing something wrong and putting the child at risk. Mothers spoke of a need for their usual life pretending that they had given birth to a healthy child.

I felt that I was going mad, I needed normality. One of the nurses recommended me to go for a walk, I went downtown for a coffee, making believe that I had not been admitted with my newborns, it felt good but at the same time I felt guilty leaving my children (ID 2629).

#### Difficulty in processing information

Parents described that it was difficult for them to process information they received about their child, and they felt that they sometimes did not have the strength to ask relevant questions. They had difficulty taking in information, and even if the medical staff told them about the risks, they did not comprehend or refused to believe that their child would not develop normally. When the staff told them that their child was doing well and in recovery, they found it hard to believe.

It was difficult for them to leave their child and go for a walk or take lunch, not knowing which nurse would take care of their child. They preferred to stay at the hospital and look after their child. When the time for discharge came, they became almost terrified and worried about what to do if they suddenly needed hospital support.

I just couldn’t process the information given by the physician, it was too much information and as soon as they told us there could be a risk of brain damage, I switched off, and just saw my little newborn, thought for myself that it couldn’t be true (ID 4183).

#### Trusting the health care system

Becoming parents of a critically sick child started an emotional turnover for the parents. A way of coping for them was their confidence in the professionals and the health care system. They described how they tried to live day by day and tried not to worry about the future. Parents said that they left the responsibility to the experts to cure their child. The confidence they felt in the health care system gave them the necessary room to grow as parents and they became relieved when they did not have to worry all the time. Trusting the health care system, information and knowledge was necessary for the parents to keep their faith that the health care system was doing everything possible to heal their child.

As time passed by, the parents became acquainted with the medical monitors, test results, and procedures at the hospital. They even thought they became experts themselves in their child’s treatment. This was experienced as challenging for them and the staff, and trustful communication was vital.

At one stage my child was critically ill, it was touch or go if he was going to survive but I did not at any time doubt that he was in the best possible hands. I felt informed and taken care of during the entire crisis (ID 6314).

### 'Getting control in a safe environment'

Parents very much looked forward to going home with their child and becoming a family. But at the same time, they had a daunting feeling about taking on the full responsibility for their child with no backup from the hospital. Once at home they started to feel safe and then they also were able to see their child and the progress the child made. The mobile application was described as a tool that gave them a sense of being in control and an active participant in the follow-up of their child.

#### Feeling safe at home

Parents felt an ultimate joy when they finally were discharged from the hospital together with their child, being able to go home to a safe and familiar environment after months in hospital. Now they could focus on their child, each other, and their new family. The child was stable and growing/developing. Getting the possibility of taking control at home in a safe environment gave the parents a feeling of overseeing their child. This was what they wished for all those months in the hospital. Their past experiences were still in the back of their minds but being surrounded by family and friends made them feel safe.

Finally, being at home after two months in hospital, I felt safe and almost ecstatic that we reached the goal of going home with our child. It also gave me the courage to develop as a mother and wife. Away from the hospital, being able to care for my child without the interference of nurses day and night, gave me a feeling of safety, it was a strange feeling but it was probably due to familiar smells and sounds that were very different from what was experienced at the hospital (ID 2896).

#### Can see that the child is developing

Coming home with their child meant a new start for the parents. Suddenly, they realized that their child was doing well, slept normally, ate well, and thrived. This brought great comfort and confidence to the parents and they sometimes they even forgot that they had had a difficult start. For some moments they even could forget about what might lie ahead. They simply looked at their child and were comforted.

After just three weeks at home I could see that my child was developing, he was now eating all his food by himself, no tube feeding, what a joy. Then a week later I got the first smile, now for sure he was developing, no doubt about it (ID 1030).

#### The app gives control and security

Parents described how the app gave them a possibility of a risk assessment for CP. Parents felt that it was better to know about the risk, and their burden was eased when they knew about their child’s risk of CP. The information leaflet about the risk of CP together with the mobile application gave them clear information. They understood that the risk was real. Understandable information helped them to deal with the uncertainty and they appreciated being able to participate in the assessment. They felt empowered in being involved and performing the assessment video, especially the fact that they themselves chose which videos were optimal for assessment, as they saw their child every day and they knew what was normal for their child.

We felt so much in control, being able to select a video that was a true picture of our child’s abilities for clinical assessment. To be honest we took five videos before uploading the video showing the true movements of our child (ID 6209).

When waiting for the result they felt anxious, describing it as a normal response when waiting for a test result. They understood it was the first stop on the recovery journey, and what was waiting for them were two roads: the low-risk road and the high-risk road. Knowing was described as better than not knowing. They had prepared themselves for both answers and were ready to deal with the result of the assessment. All parents, without exception described that they felt secure during the waiting time and that they felt fully informed about how and when they were going to be informed about the result. They were aware of the direct line to the principal investigator, which meant that they could get advice during the waiting time for the result. Not knowing was described as worrying. Not if the result would be ‘high-risk’ or ‘low-risk'.

By having the In-motion app on their smartphone, the parents were able to talk to their family and friends about their child’s follow-up, which gave them confidence and prepared them for their upcoming outpatient meetings with the neonatologist and physiotherapist. They were able to prepare questions in advance and thereby get a more profound understanding of how their child’s future would be.

For the first time I felt as an active partner in the treatment of my child. I was well prepared for the outpatient appointment with my child’s doctor and physiotherapist and felt that the treatment plan for the future was just as much my plan as the plan decided by the medical professionals. I’ve got answers to all my questions; what lovely sentiment (ID 2896).

## Discussion

Qualitative interviews with parents showed that the early risk assessment for CP using a mobile application did not seem to worry the parents but gave them instead a kind of security. The risk assessment they performed by themselves through the mobile application was experienced as rewarding and helpful to them after the strain caused by the long stay in the hospital. During their hospitalization they experienced no control and had to cope with different information from a variety of health care professionals at all hours of the day. Being at home, the parents appreciated the concise information about how to use the application and they appreciated being involved in the plan after discharge when they knew what was expected of them.

The parents’ experiences during their hospital stay are similar to what is described in earlier research [21]. Contradictory information and guidelines from different healthcare professionals and information they did not understand or could not cope with worried the parents and gave them a sense of an uncontrolled life situation. Kowalski et al. [22] likewise found that although the parents were informed about risk factors, 20–30% of all parents said they were not sufficiently informed about the risk of permanent neurological damage. The communication between parents and healthcare professionals is vital for parents to cope when they are hospitalized with a severely ill preterm born child. Parents in earlier studies described the communication as either positive or negative, depending on how much they felt that the information helped them to cope with the situation [23]. Several other studies have also shown the need for transparency for parents to feel safe and empowered [24–26].

The discharge for which parents prepared themselves in our study was both a long-awaited day and a day associated with worry, knowing that they would have to care for their child themselves. Most of all it felt like a happy day, going home, and settling into their own routines, and trying to find control in their lives at home. For the first time they felt as a family, finally being able to grow as a family on their own accord. The successful discharge and the easily understood information for the app was important for their use of the application at home and for their sense of security in using the application. The application entailed that the same information was given to all parents, both orally and visually through an instruction video and written information with links to evidence-based medical research. They knew exactly when and how to record the video and why they had to record their child’s movement. Giving parents scientific knowledge makes them strong as parents and an equal partner to the health care professionals in the follow-up of their child [22,23]. On the other hand, Wigert et al. [27] showed that the lack of accurate communication could give the parents feelings of loneliness, abandonment, or unwanted responsibility.

Communication about early diagnosis or high risk for CP is vital for both parents and health care providers; however, some parents find healthcare providers overall pessimistic [23,28]. Parents request information and education surrounding their child’s early risk assessment or early diagnosis [23,29,30]. In that context our study shows strong elements of communication and education as the parents were receiving information both orally and written; besides that, they had a lifeline where they could get in contact with a health professional.

We used the In-Motion app developed in Norway. The Baby Moves app is similar [14] to the one used in our study. The two apps are similar, the main difference between the two is that the In-Motion app has an instruction video that the parents must watch prior to recording their child with the app. The In-Motion was likewise designed to be used in connection with computer-based GM assessment, which demands that the video is recorded in a specific way [15]. However, both apps are only available for research.

By using the application, the parents in our study decided when and which video recording was going to be used for the assessment. This meant that the parents were more likely to record their child in the right behavioral state which is required by the GMA method [10]. Parents knew what was going to happen at the follow-up appointment and described that for the first time they had a plan to stick to. Engaging the parents actively in the follow-up of their child empowered them in their further engagement in their child’s care and treatment. Lakshmanan et al. [31] showed that parents emphasized that mobile health technology would be helpful in preparing for both discharge and follow-up; they explained that all the information about the follow-up was gathered in one device which made life so much easier, besides which they could do it without going into hospital. Transferring the video recording for early assessment for cerebral palsy from the hospital to a safe home environment may help parents to feel empowered and gain control over their child’s follow-up. However, it is important to find technical solutions that are both easy and safe to use for all involved, easily accessible for families, and efficient to use for the professionals. When developing and evaluating new eHealth solutions, the participation of end users is vital [14,15].

In our study, information about GMA, CP and parents support groups and local services is part of the Danish healthcare system for all infants. The parents in our study were pleased using the In-Motion app for early risk assessment for CP and by knowing that they could contact health professionals until they received the result from the videorecording which probably increased their feelings of security. Early diagnosis or early risk assessment for CP is considered golden standard [8]. Research demonstrates that there is overall agreement between parents and healthcare providers that early diagnosis or early risk assessment is vital for long-term outcome and facilitates an open dialogue about the future of the child [32]. Parents also request written material regarding CP, support groups and local services [29,30] to support them.

### Strengths and limitations

Qualitative interviews and a hermeneutic phenomenological approach as described by van Manen [18,19] is proved to be well suited to elucidate parents’ lived experience. The authors all came into the study with a different preunderstanding which is considered a strength. The first author, Annemette Brown, is a pediatric physiotherapist with many years’ experiences in the field of early prediction of adverse neurological development in children with high risk, and in working with parents in stressful situations like this. She was working at one of the neonatal wards where the infants were cared for but was not involved in the care of the infants. The second author, Åsa B. Tornberg, is an associate professor of physiotherapy with many years’ experiences in clinical research. The last author, Inger Kristensson Hallström, is a professor of pediatric nursing with extensive experience of sick children and their families as well as in qualitative research and especially with-in the hermeneutic phenomenology by van Manen. The authors’ preunderstanding was discussed and made explicit throughout the analyses and writing processes so that their preunderstanding did not influence the results in a subjective way.

To support the arguments for early intervention, our project planning, data and interpretations were dealt with at seminars arranged with fellow researchers having a multidisciplinary background as recommended by van Manen [18,19]. To ensure confirmability and let parents’ voices be reflected in our findings, the study aimed to represent their lived experiences as gained first-hand, without any reflection or analysis [18,19]. The subjective and unique experience of each parent is shown by quotations in the text. Dependability was ensured by adopting a line-by-line approach, a systematic presentation of data and arrangement of the study process, and by having three researchers with different backgrounds analyze the data [33].

A limitation of the study was the homogeneity of the parents; they were either Danish or English speaking and none came from non-western countries. Further, all the parents came from one health region in Denmark. This implies that the results might not be transferable to parents in other contexts or other health regions and countries.

We followed the recommendations from van Manen [18,19], and parents did not review the transcribed manuscripts, nor did they get a chance to comment on the themes and subthemes. However, the parents’ views could have strengthened the trustworthiness of the results.

## Conclusion

After giving birth to a child at risk of CP parents struggle with loss of control and anxiety about the future. By using a mobile application for video recordings by the parents at home at a time which suits the child and the family it is possible to empower and involve parents in the care of their newborn child. In the future, remote video recording for general movement assessment in clinical practice is a means to ensure early identification of the risk of developing cerebral palsy in a high-risk population and thereby enable early interventions. However, a digital platform that can be used in clinical practice for safe transmission of videos is needed. A platform where parents additionally can get information of the newest research and the possibility of communicating with healthcare personnel or get in touch with parent support groups.

## Data Availability

The data are not publicly available due to the nature thereof: the interviews are very personal, and access is therefore restricted due to confidentiality. We summarized the data of the participants in the manuscript text. The data of the findings in this study can be provided from PhD student Annemette Brown on reasonable request.
